# Distribution of neocarzinostatin conjugated to biotinylated chimeric monoclonal antibody Fab fragments after administration of avidin.

**DOI:** 10.1038/bjc.1996.407

**Published:** 1996-08

**Authors:** E. Otsuji, T. Yamaguchi, H. Matsumura, K. Yamamoto, H. Tsuruta, Y. Yata, H. Nishi, K. Okamoto, K. Kitamura, T. Takahashi

**Affiliations:** First Department of Surgery, Kyoto Prefectural University of Medicine, Japan.

## Abstract

We have developed chimeric Fab fragments of MAb A7 (chA7Fab) and have reported on their potential usefulness as a carrier of neocarzinostatin (NCS). However, a large amount of chA7Fab accumulates in the kidneys which might cause renal failure. This was one of the major side-effects of the chA7Fab-NCS immunoconjugate administered to humans. To decrease the kidney accumulation of chA7Fab, chA7Fab was biotinylated and administered with a subsequent injection of avidin to nude mice with pancreatic cancer. The accumulation of biotinylated chA7Fab in the blood and the kidneys decreased significantly after the injection of avidin. In a separate experiment with biotinylated chA7Fab-NCS, the blood and kidney accumulation decreased significantly after the injection of avidin. These findings suggest that the injection of biotinylated chA7Fab complexed with NCS followed by avidin may be safer and may permit the administration of larger doses of NCS without the subsequent development of renal failure.


**British Journal of Cancer (1996) 74, 597-602**
© 1996 Stockton Press All rights reserved 0007-0920/96 $12.00

### Distribution of neocarzinostatin conjugated to biotinylated chimeric monoclonal antibody Fab fragments after adminstration of avidin

E Otsuji, T Yamaguchi, H Matsumura, K Yamamoto, H Tsuruta, Y Yata, H Nishi, K Okamoto, K Kitamura and T Takahashi

*First Department of Surgery, Kyoto Prefectural University of Medicine, Kamigyo-ku, Kyoto 602, Japan.*

**Summary** We have developed chimeric Fab fragments of MAb A7 (chA7Fab) and have reported on their potential usefulness as a carrier of neocarzinostatin (NCS). However, a large amount of chA7Fab accumulates in the kidneys which might cause renal failure. This was one of the major side-effects of the chA7Fab-NCS immunoconjugate administered to humans. To decrease the kidney accumulation of chA7Fab, chA7Fab was biotinylated and administered with a subsequent injection of avidin to nude mice with pancreatic cancer. The accumulation of biotinylated chA7Fab in the blood and the kidneys decreased significantly after the injection of avidin. In a separate experiment with biotinylated chA7Fab-NCS, the blood and kidney accumulation decreased significantly after the injection of avidin. These findings suggest that the injection of biotinylated chA7Fab complexed with NCS followed by avidin may be safer and may permit the administration of larger doses of NCS without the subsequent development of renal failure.

**Keywords:** biotinylated antibody; chimeric antibody; avidin; targeting chemotherapy; monoclonal antibody A7; pancreatic cancer

### Introduction

Pancreatic cancer is one of the most lethal of all cancers. One of the reasons for its high mortality is the lack of effective chemotherapy. A number of murine monoclonal antibodies (MAbs) have been linked to various anti-tumour drugs, cytotoxins and enzymes in an attempt to increase the effectiveness of chemotherapy (Apelgren *et al.*, 1990). We have also produced MAb A7, which reacts with pancreatic and colonic carcinomas, from human colonic carcinomas, and covalently conjugated it with the anti-cancer drug, neocarzinostatin (NCS) (Fukuda, 1985). A7-NCS has been used clinically for the treatment of patients with advanced colorectal and pancreatic cancers. However, human anti-mouse antibody (HAMA) was detected in all patients who received A7-NCS (Takahashi *et al.*, 1993). HAMA usually increases the clearance of the administered MAb, thus reducing the MAb tumour accumulation and possibly resulting in lower therapeutic efficacy of the MAb-drug conjugate.

In Fujita's previous study, more than 70% of the NCS was inactivated by the serum within 120 min (Fujita *et al.*, 1970). In general, because the Fab fragments of MAbs are able to penetrate target tumours, they may be more suitable as carriers of anti-cancer agents such as NCS which are inactivated rapidly in the blood.

For these reasons, we have developed chimeric Fab fragments of MAb A7 (chA7Fab) (Yamaguchi *et al.*, 1993) as a carrier of NCS. In a previous study using nude mice bearing pancreatic carcinoma (which reacts with MAb A7), a larger amount of $^{125}$I-labelled chA7Fab accumulated in the pancreatic carcinoma compared with that of $^{125}$I-labelled MAb A7. Regarding normal tissue accumulation, however, more chA7Fab than $^{125}$I-labelled MAb A7 accumulated in the kidney because most of the intravenously injected chA7Fab was cleared via the kidney. Because renal dysfunction is one of the major side-effects of NCS chemotherapy in human, the kidney accumulation of chA7Fab should be minimised when it is administered as a carrier of NCS.

The association constant of the avidin-biotin binding is approximately $10^{15}$ M$^{-1}$, and much greater than that of antigen-antibody interactions (Green, 1963). Because avidin has four biotin-binding sites per molecule, aggregates formed by avidin and biotinylated antibody are produced if avidin is injected following the administration of biotinylated antibody. Once such aggregates are formed in the body, they could be taken up rapidly by the reticuloendothelial system and their accumulation in the blood and kidney should decrease accordingly. In this study we investigated the *in vivo* distribution of biotinylated chA7Fab and biotinylated chA7Fab-NCS after the administration of avidin which appeared to decrease the accumulation of chA7Fab in the kidney.

### Materials and methods

### *Cell line and tumour xenografts*

The human pancreatic carcinoma cell line, HPC-YS, used in this study was established from a ductal cell adenocarcinoma of the human pancreas and was obtained from N Yamaguchi (Research Institute of Neurology and Geriatrics, Kyoto Prefectural University of Medicine, Japan) (Otsuji *et al.*, 1993). HPC-YS cells were maintained in RPMI-1640 medium supplemented with 10% fetal bovine serum (FBS) (Flow Laboratories Inc., Rockville, MD, USA). Cultured HPC-YS cells were harvested by brief treatment with EDTA, washed in phosphate-buffered saline (PBS) and resuspended in PBS. Approximately $5 \times 10^6$ viable cells were injected subcutaneously (s.c.) into the left flank of athymic 8-week-old male mice (BALB/C, nu-nu) (SLC Co., Shizuoka, Japan). A tumour mass detected in all the mice injected with the HPC-YS cells. These mice were treated humanely according to the guidelines in our Institute.

### *Preparation and purification of chA7Fab*

Murine MAb A7 is an $IgG_1$ and has been reported to react with 77% of human pancreatic carcinomas tested, as well as with 70% of the human colonic carcinomas tested (Otsuji *et al.*, 1993). MAb A7 does not react immunohistochemically with normal pancreatic tissues (Otsuji *et al.*, 1990). ChA7Fab was produced as described previously (Yamaguchi *et al.*,

Correspondence: E Otsuji, First Department of Surgery, Kyoto Prefectural University of Medicine, Kawaramachi Hirokoji Kamigyoku, Kyoto 602, Japan
Received 1 September 1995; revised 5 February 1996; accepted 14 March 1996

**Figure 1** The accumulation of $^{125}$I-labelled biotinylated chA7Fab in the blood, HPC-YS tumours and the normal tissues of mice with or without the subsequent administration of avidin (**a**, blood; **b**, tumour; **c**, heart; **d**, liver; **e**, spleen; **f**, pancreas; **g**, colon; **h**, kidney). The mice received an injection of avidin in 100 $\mu$l of 50 mM HEPES in 5% mannitol buffer or the same solution without avidin, respectively, at 1, 6, 12 and 24 h after the injection of $^{125}$I-labelled biotinylated chA7Fab. The pattern of tumour accumulation of $^{125}$I-labelled biotinylated chA7Fab with the subsequent administration of avidin was similar to that without administration of avidin. The blood concentration of $^{125}$I-labelled biotinylated chA7Fab was significantly lower following the administration of avidin than without its administration at 1 and 6 h after the injection of $^{125}$I-labelled biotinylated chA7Fab. The kidney accumulation of $^{125}$I-labelled biotinylated chA7Fab was significantly lower with the subsequent administration of avidin than it was without the administration of avidin at 1 and 6 h after antibody injection. The accumulation of $^{125}$I-labelled biotinylated

1993). Briefly, a murine light-chain variable region gene was joined to a human $\kappa$ light-chain constant region gene. A murine heavy-chain variable region gene was joined to a human $\gamma_1$ heavy-chain constant region gene to construct a human–mouse chimeric heavy-chain gene. The plasmid DNAs were introduced into AH22 yeast cells as described previously. After incubation in YPD medium for 3 days, the cellular debris was removed from the medium by centrifugation and purified using a CM Sepharose 4B anti-human IgG column.

### *Neocarzinostatin conjugation to chA7Fab*

ChA7Fab was conjugated to NCS (Kayaku, Tokyo, Japan) with *N*-succinimidyl-3-(2-pyridyldithio)-propionate (SPDP) as described previously by Yamaguchi *et al.* (1993). The conjugation ratio was 1 mol of NCS per mol of chA7Fab.

### *Preparation of radiolabelled chA7Fab and chA7Fab–NCS*

Radiolabelling of chA7Fab with $^{125}$I (Amersham Japan Ltd., IMS 30, Japan) was performed by the chloramine-T method (Hunter and Greenwood, 1962). Iodinised chA7Fab was separated from unconjugated reagents by gel filtration on a Sephadex G-25 column. ChA7Fab–NCS was radiolabelled with $^{125}$I by the same method. ChA7Fab and chA7Fab–NCS was labelled with $^{125}$I to a specific activity of 4.5 $\mu$Ci $\mu g^{-1}$ and 4.2 $\mu$Ci $\mu g^{-1}$ respectively.

### *Biotinylation of $^{125}$I-labelled chA7Fab and $^{125}$I-labelled chA7Fab–NCS*

NHS–biotin (180 $\mu$g) (Pierce 23225, IL, USA) in dimethyl sulphoxide (DMSO) was added to 120 $\mu$g of $^{125}$I-labelled chA7Fab in 50 mM sodium bicarbonate buffer, pH 8.5. Following incubation on ice for 90 min, the preparations were incubated for 30 min at room temperature. $^{125}$I-labelled biotinylated chA7Fab was separated from unreacted biotin by gel filtration on a Sephadex G-25 column. $^{125}$I-labelled biotinylated chA7Fab–NCS was also biotinylated by the same method. $^{125}$I-labelled biotinylated chA7Fab and $^{125}$I-labelled biotinylated chA7Fab–NCS was stored at 4°C in 0.1 M sodium phosphate, pH 7.0 until use.

### *Biodistribution of $^{125}$I-labelled chA7Fab and $^{125}$I-labelled chA7Fab–NCS after administration of avidin in nude mice bearing tumours*

The distribution of $^{125}$I-labelled biotinylated chA7Fab after the administration of avidin was investigated in athymic nude mice bearing HPC-YS tumours. Fourteen days after inoculation, the tumour-grafted mice were divided into two groups. Thirty-two mice were divided into two groups and mice in both groups injected intravenously with 0.7 $\mu$Ci of $^{125}$I-labelled biotinylated chA7Fab in 100 $\mu$l of PBS. Four mice from each group were injected intravenously with 30 $\mu$g of streptavidin (Sigma, MO, USA) in 100 $\mu$l of 50 $\mu$M HEPES in 5% mannitol buffer, pH 7.4 or the same solution without streptavidin respectively at 1, 6, 12 and 24 h after the injection of $^{125}$I-labelled biotinylated chA7Fab. They were killed 30 min later and dissected. After dissection, the tumours, blood and normal organs (heart, liver, spleen, pancreas, colon and kidney) were weighed. The mean weight of the tumours was 145 mg. The radioactivity in each tissue was then measured using a $\gamma$-scintillation counter (Auto-Gamma, 5000, Packard). The results from the various tissues were expressed as c.p.m. $g^{-1}$ and compared with each other. To compare the kinetics of $^{125}$I-labelled biotinylated chA7Fab in each group, the results were presented as %ID $g^{-1}$ (% injected dose of radioactivity per g). The distribution of $^{125}$I-labelled biotinylated chA7Fab–NCS after the administration of avidin was also examined in athymic nude mice bearing HPC-YS tumours. $^{125}$I-labelled biotinylated chA7Fab–NCS was injected with or without the subsequent administration of streptavidin and tumours, blood and normal organs (heart, liver, spleen, pancreas, colon and kidney) were collected. The weight and radioactivity of each tissue was measured and then the results were presented as %ID $g^{-1}$. Student's *t*-test was used to check for statistically significant differences.

### Results

### *Biodistribution of $^{125}$I-labelled chA7Fab after administration of avidin in nude mice bearing tumours*

The pattern of tumour accumulation of $^{125}$I-labelled biotinylated chA7Fab with the subsequent administration of avidin was similar to that when avidin was not administered. The tumour accumulation of $^{125}$I-labelled biotinylated chA7Fab with and without the administration of avidin decreased linearly with time. In contrast, the concentration of $^{125}$I-labelled biotinylated chA7Fab in the blood was lower with the administration of avidin than without it. Significant differences were observed between the two groups at 1 and 6 h after injection ($P<0.05$). As for normal tissues, the kidney accumulation of $^{125}$I-labelled biotinylated chA7Fab was lower with the administration of avidin than that without it. Significant differences were observed between the two groups at 1 and 6 h after injection ($P<0.05$). The accumulation of $^{125}$I-labelled biotinylated chA7Fab in the spleen and the liver was higher with the administration of avidin than that without it. Significant differences were observed between the two groups at 1 and 6 h after injection for the spleen and the liver ($P<0.05$) (Figure 1).

### *Biodistribution of $^{125}$I-labelled chA7Fab–NCS after administration of avidin in nude mice bearing tumours*

The tumour accumulation of $^{125}$I-labelled biotinylated chA7Fab–NCS with the administration of avidin was almost identical with that without administration of avidin. In contrast, the concentration of $^{125}$I-labelled biotinylated ch7Fab–NCS in the blood was lower with the administration of avidin than without it. Significant differences were observed between the two groups at 1, 6 and 12 h after injection ($P<0.05$). The kidney accumulation of $^{125}$I-labelled biotinylated chA7Fab–NCS was lower with the administration of avidin than that without it. Significant differences were observed between the two groups at 1 and 6 h after injection ($P<0.05$). The accumulation of $^{125}$I-labelled biotinylated chA7Fab–NCS in the spleen and the liver was higher with the administration of avidin than that without it. Significant differences were observed between the two groups at 1 and 6 h after injection for the spleen and at 1 h for the liver ($P<0.05$) (Figure 2).

### Discussion

We have reported that MAb A7 can be linked covalently to NCS and that this conjugate can be used to treat patients with colorectal cancer. Although some of the patients who chA7Fab in the spleen and the liver was higher following the administration of avidin than it was without the administration of avidin. Significant differences were observed between the two groups at 1 and 6 h after the antibody injection for the spleen and the liver. A, with the subsequent administration of avidin; B, no avidin administration; point, mean; bar, s.d.; *, significant difference ($P<0.05$).

**Figure 2** The accumulation of $^{125}$I-labelled biotinylated chA7Fab–NCS in the blood, HPC-YS tumours and the normal tissues of mice with or without the subsequent administration of avidin (**a**, blood; **b**, tumour; **c**, heart; **d**, liver; **e**, spleen; **f**, pancreas; **g**, colon; **h**, kidney). The mice received an injection of avidin in 100 $\mu$l of 50 mM HEPES in 5% mannitol buffer or the same solution without avidin, respectively, at 1, 6, 12 and 24 h after the injection of $^{125}$I-labelled biotinylated chA7Fab–NCS. The pattern of the accumulation of $^{125}$I-labelled biotinylated chA7Fab–NCS in each tissue with or without the subsequent administration of avidin was similar to that of $^{125}$I-labelled biotinylated chA7Fab. A, with the subsequent administration of avidin; B, no avidin administration; point, mean; bar, s.d.; *, significant difference ($P<0.05$).

have been treated with A7–NCS have had a partial regression of their tumours, HAMA was produced in all patients who received A7–NCS (Takahashi *et al.*, 1993). HAMA production should decrease when NCS conjugated to a human/mouse chimeric MAb is administered to humans, because the origin of the Fc portion of the chimeric MAb, which is the most immunopotent region of intact MAbs (Spiegelberg *et al.*, 1965), is human. Fujita *et al.* (1970) reported that more than 70% of the anti-tumour activity of NCS was inactivated by 10% mouse serum within 120 min in an *in vitro* experiment. In general, the small variable fragment of the MAb molecule has the ability to leave the vascular space rapidly and to penetrate easily into target tumour tissue. Thus, Fab fragments of MAb A7 may be suitable as carriers of anti-cancer agents like NCS which are inactivated rapidly in the blood. For these reasons, we have prepared chA7Fab and have conjugated it to NCS. ChA7Fab specifically accumulated in the tumour (Otsuji *et al.*, 1995) and the conjugate showed greater anti-tumour activity against human pancreatic cancer growth in nude mice than did A7–NCS and completely suppressed tumour growth (Otsuji *et al.*, 1996). However, a large amount of chA7Fab accumulated in the kidney, consistent with the results of Hansson *et al.* (1988) who have demonstrated the rapid clearance of non-Fc-bearing antibody fragments. This clearance is thought to occur mainly via the kidney. Because renal dysfunction is one of the most lethal adverse effects of NCS in humans, it would seem prudent to reduce the kidney accumulation of chA7Fab, conjugated to NCS.

Avidin, a 66 kDa protein found in egg whites, has a strong avidity for biotin, a 244 Da vitamin found in low concentrations in tissues and in blood (Mock and Dubois, 1986). The association constant of the avidin–biotin bond is $10^{15}$ $M^{-1}$ (Green, 1963) and, as such, is $10^6$-fold greater than most antigen-antibody interactions. The bond formation is completed within 15 min and, once formed, the bond is extremely stable. The *in vitro* method of biotinylation of proteins has been well described, and any type of immunoglobulin can be biotinylated easily.

The strong affinity of the avidin–biotin system has drawn the attention of several researchers working on background reduction in the imaging applications of MAbs (Hnatowich *et al.*, 1987; Sinitsyn *et al.*, 1989; Ogihara *et al.*, 1994). This is thought to be secondary to the trapping by the reticuloendothelial system of avidin–biotinylated MAb aggregates formed in the serum. These results suggested that avidin might be useful in targeting NCS conjugated to chA7Fab away from the kidneys. As shown in Figure 2a, a significantly smaller amount of biotinylated chA7Fab–NCS is retained in the blood in the group of mice injected with avidin compared with those not injected with avidin. In a previous study, $^{125}$I-labelled chA7Fab–NCS was cleared from the blood via the kidney and a large amount of chA7Fab–NCS accumulated in the kidney (Otsuji *et al.*, 1994). As shown in Figure 2h, the kidney concentration of biotinylated chA7Fab–NCS decreased approximately 50% by the subsequent administration of avidin. Decreased renal dysfunction can be expected by the administration of biotinylated chA7Fab–NCS along with avidin. As for the tumours, the accumulation of biotinylated chA7Fab–NCS was not affected by the administration of avidin, while it did significantly increase the accumulation of biotinylated chA7Fab–NCS in the spleen and the liver. However, the splenic accumulation of biotinylated chA7Fab conjugated to NCS is unlikely to be toxic to patients because there have been no reports of adverse effects of NCS on splenic function. Although a relatively large amount of biotinylated chA7Fab–NCS also accumulated in the liver, NCS is reported to be inactivated rapidly in the liver (Fujita *et al.*, 1970), which may minimise its toxicity. In another experiment, the distribution of biotinylated chA7Fab with a subsequent injection of avidin was similar to that of biotinylated chA7Fab–NCS. In the separate experiments using unbiotinylated chA7Fab with a subsequent injection of avidin, the distribution of unbiotinylated chA7Fab was identical to that without injection of avidin (data not shown). Therefore, this high accumulation of biotinylated chA7Fab–NCS in the spleen and the liver was considered due to the trapping of the avidin–biotinylated chA7Fab aggregates by the reticuloendothelial system.

Because avidin–biotinylated MAb aggregates were formed in the serum, the immunogenity of this complex might be enhanced. However, with the avidin doses used in this study, we did not observe any side-effects in the animals. Other researchers have also reported that such doses of avidin are not toxic (Hnatowich *et al.*, 1987; Ogihara *et al.*, 1994; Paganelli *et al.*, 1990).

From these results, we conclude that the accumulation of biotinylated chA7Fab–NCS in the blood and the kidneys can be decreased by the subsequent administration of avidin and this modification does not reduce tumour accumulation. Thus, biotinylated chA7Fab may be a suitable carrier of NCS and result in less kidney toxicity if avidin is also administered, preferably between 1 and 6 h after the administration of antibody conjugate.

**Acknowledgement**
This work was supported in part by a Grant-in-Aid from the Pancreatic Research Foundation of Japan.

### References

APELGREN LD, ZIMMERMAN DL, BRIGGS SL AND BUMOL TF. (1990). Antitumour activity of monoclonal antibody–Vinca alkaloid immunoconjugate LY203725 (KS1/4-4-desacetylvinblastine-3-carboxyhydrazide) in a nude mouse model of human ovarian cancer. *Cancer Res.*, **50**, 3540–3544.

FUJITA H, NAKAYAMA N, SAWABE T AND KIMURA K. (1970). *In vivo* distribution and inactivation of neocarzinostatin. *Jpn. J. Antibiotics*, **23**, 471–478.

FUKUDA K. (1985). The study of targeting chemotherapy against gastrointestinal cancer. *Akita J. Med.*, **12**, 451–468.

GREEN NM. (1963). The use of [$^{14}$C] biotin for kinetic studies and for assay. *Biochem. J.*, **89**, 585–591.

HANSSON Y, PAULIE S, BEN-AISSA H, RUDBERG U, KARLSSON A AND PERLMANN P. (1988). Radioimmunolocalization of bladder tumors xenotransplanted in nude mice. *Anticancer Res.*, **8**, 435–442.

HNATOWICH DJ, VIRZI F AND RUSCKOWSKI M. (1987). Investigations of avidin and biotin for imaging applications. *J. Nucl. Med.*, **28**, 1294–1302.

HUNTER WM AND GREENWOOD FC. (1962). Preparation of iodine, $^{131}$I-labelled human growth hormone of high specific activity. *Nature*, **194**, 495–496.

MOCK DM AND DUBOIS DB. (1986). A sequential, solid-phase assay for biotin in physiologic fluids that correlates with expected biotin status. *Ann. Biochem.*, **153**, 272–278.

OGIHARA I, SASAKI T, TOYAMA H, ODA K, SENDA M AND NISHIGORI H. (1994). Rapid tumor imaging by active background reduction using biotin-bearing liposomes and avidin. *Cancer Res.*, **54**, 463–467.

OTSUJI E, TAKAHASHI T, YAMAGUCHI T, YAMAGUCHI N AND IMANISHI J. (1990). Specific cytotoxic effect of neocarzinostatin conjugated to monoclonal antibody A7 on human pancreatic carcinoma. *Gastroenterol. Jpn.*, **25**, 244–248.

OTSUJI E, YAMAGUCHI T, YAMAOKA N, KITAMURA K, YAMAGUCHI N, IMANISHI J AND TAKAHASHI T. (1993). Increased antitumor effect of neocarzinostatin conjugated to monoclonal antibody A7 on human pancreatic carcinoma grafted in nude mice. *Antibody, Immunoconjugates and Radiopharmaceuticals*, **6**, 177–183.

OTSUJI E, YAMAGUCHI T, YAMAOKA N, TANIGUCHI K, KATO M, KOTANI T, KITAMURA K AND TAKAHASHI T. (1994). Biodistribution of neocarzinostatin conjugated to chimeric Fab fragments of the monoclonal antibody A7 in nude mice bearing human pancreatic cancer xenografts. *Jpn. J. Cancer Res.*, **85,** 530–535.

OTSUJI E, YAMAGUCHI T, YAMAOKA N, KOTANI T, KATO M, TANIGUCHI T, KITAMURA K AND TAKAHASHI T. (1995). Biodistribution of murine and chimeric Fab fragments of the monoclonal antibody A7 in human pancreatic cancer. *Pancreas*, **10,** 265–273.

OTSUJI E, YAMAGUCHI T, TSURUTA H, YATA Y, NISHI H, OKAMOTO K, TANIGUCHI K, KATO M, KOTANI T, KITAMURA K AND TAKAHASHI T. (1996). Effects of neocarzinostatin–chimeric Fab conjugates on the growth of human pancreatic carcinoma xenografts. *Br. J. Cancer*, (in press).

PAGANELLI G, PERVES S, SICCARDI AG, ROWLINSON G, DELEIDE G, CHIOLERIO F, MALCOVATI M, SCASSELLATI, GA AND EPENETOS AA. (1990). Intraperitoneal radio-localization of tumors pre-targeted by biotinylated monoclonal antibodies. *Int. J. Cancer*, **45,** 1184–1189.

SINITSYN VV, MAMONTOVA AG, CHECKNEVA YY, SHNYRA AA AND DOMOGATSKY SP. (1989). Rapid blood clearance of biotinylated IgG after infusion of avidin. *J. Nucl. Med.*, **30,** 66–69.

SPIEGELBERG HL AND WEIGLE WO. (1965). The catabolism of homologous and heterologous 7s gamma globulin fragments. *J. Exp. Med.*, **121,** 323–338.

TAKAHASHI T, YAMAGUCHI T, KITAMURA K, NOGUCHI A, HONDA M AND OTSUJI E. (1993). Follow-up study of patients treated with monoclonal antibody–drug conjugate: report of 77 cases with colorectal cancer. *Jpn. J. Cancer Res.*, **84,** 976–981.

YAMAGUCHI T, TSURUMI H, KITAMURA K, OTSUJI E, MIYAGAKI T, KOTANI T AND TAKAHASHI T. (1993). Production, binding and cytotoxicity of human/mouse chimeric monoclonal antibody–neocarzinostatin conjugate. *Jpn. J. Cancer Res.*, **84,** 1190–1194.


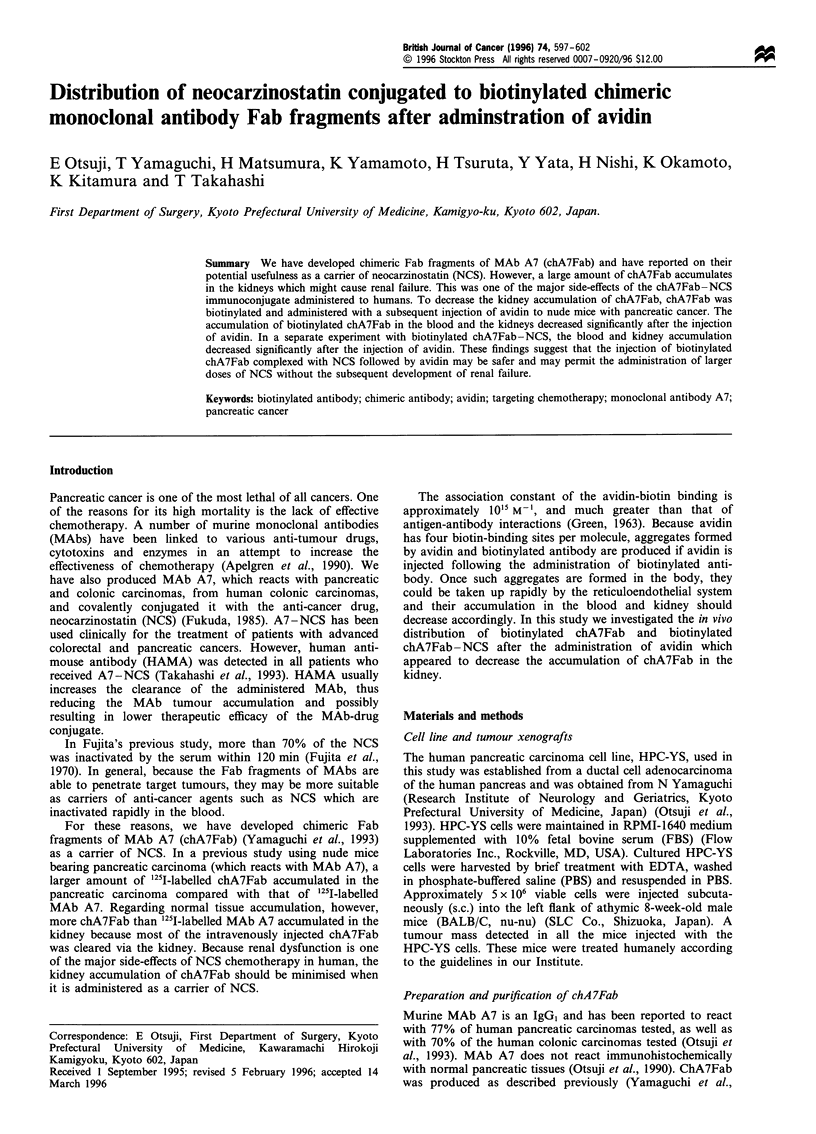

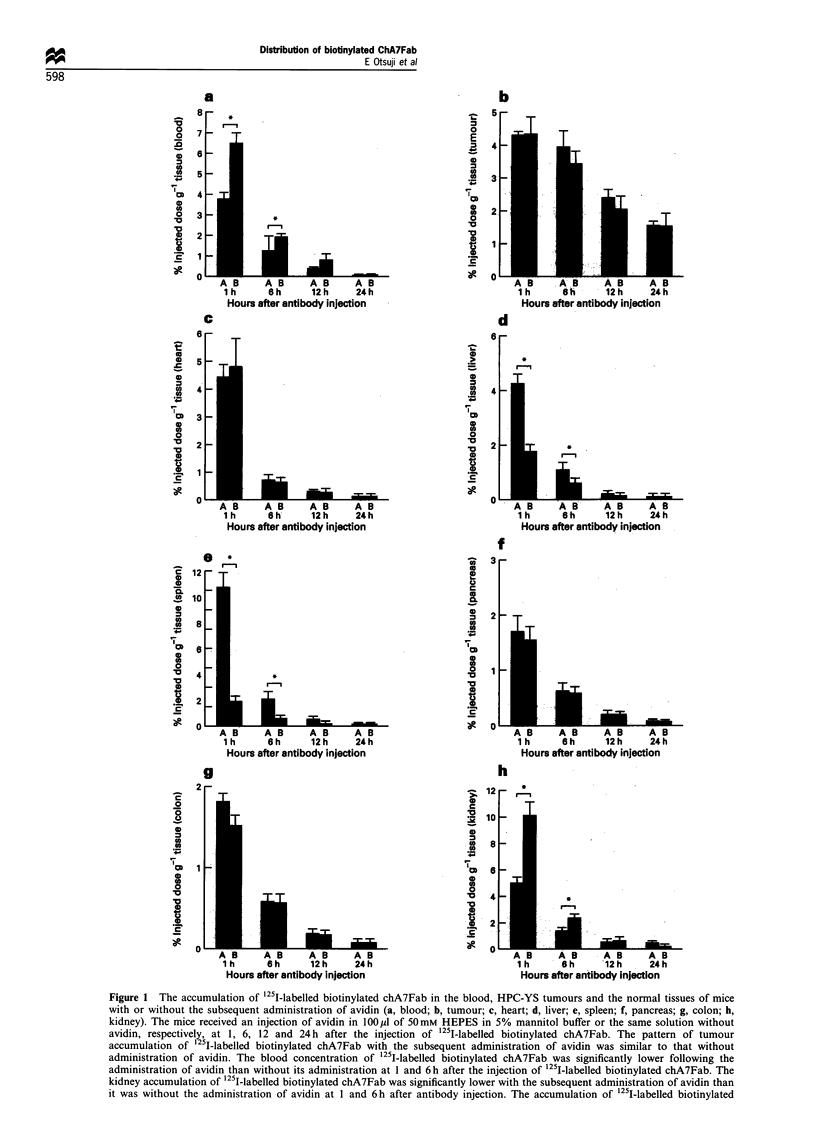

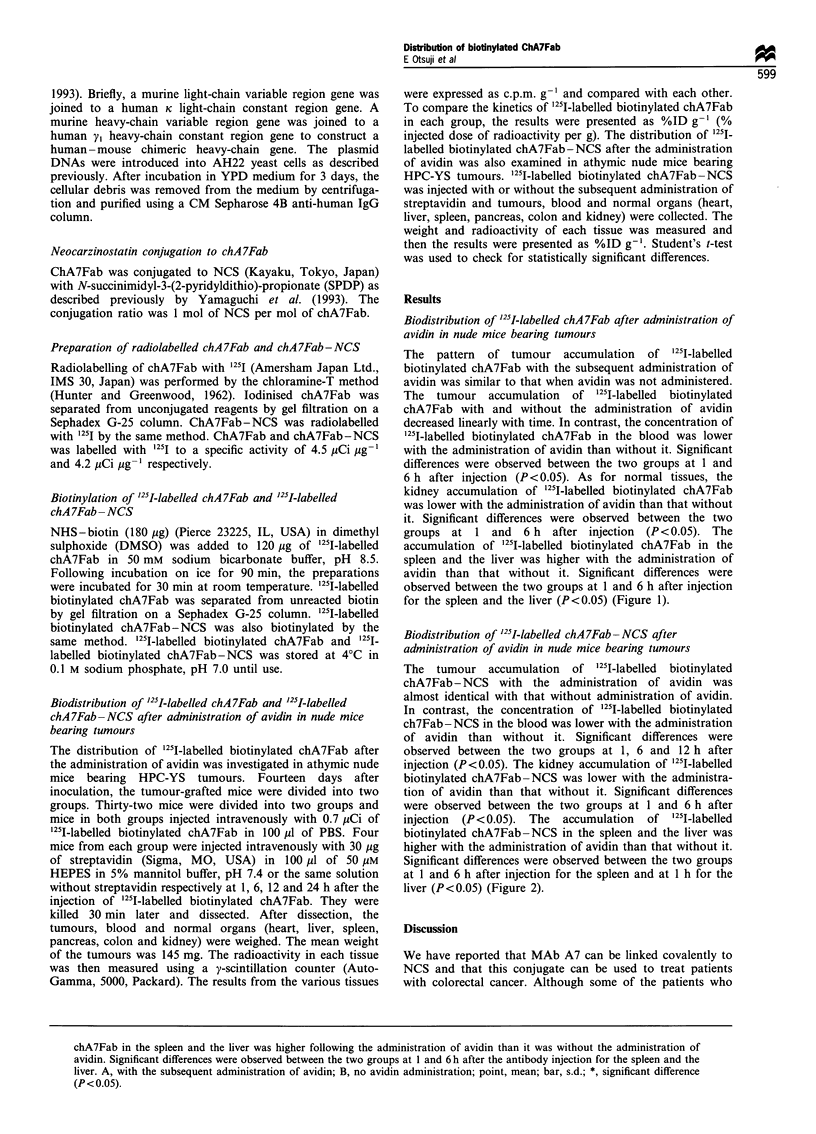

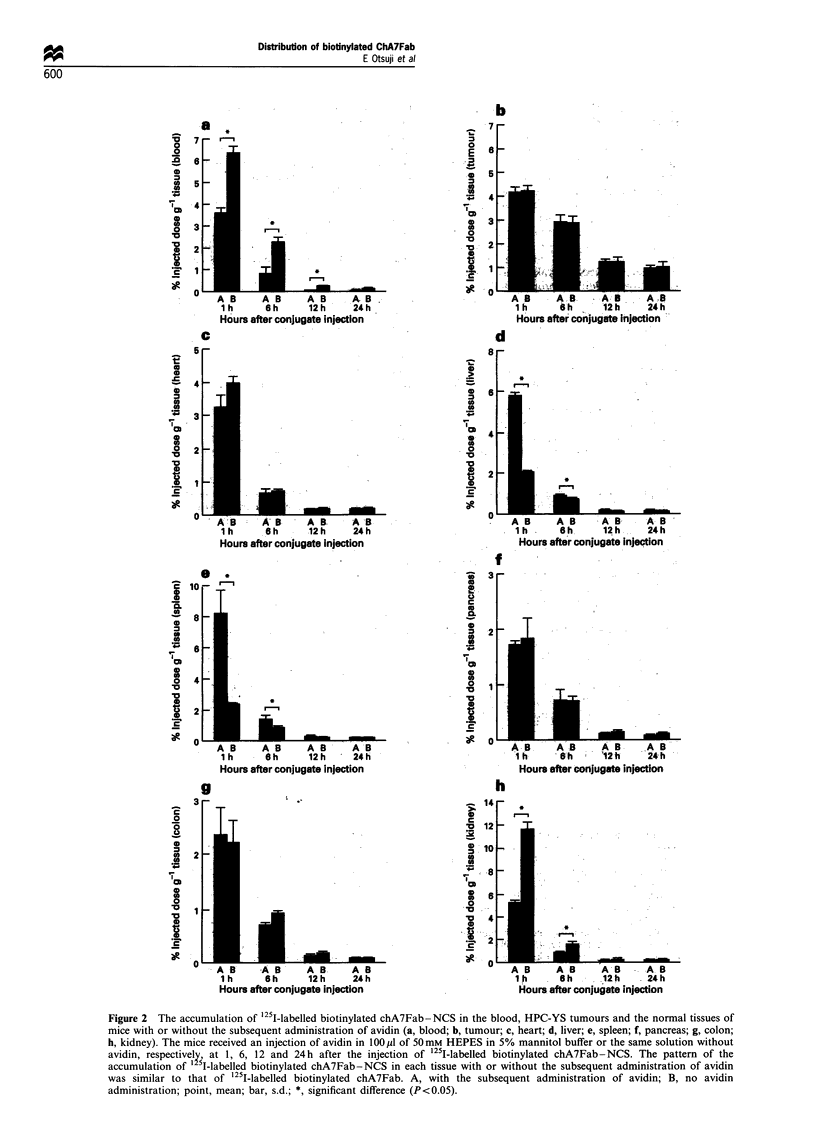

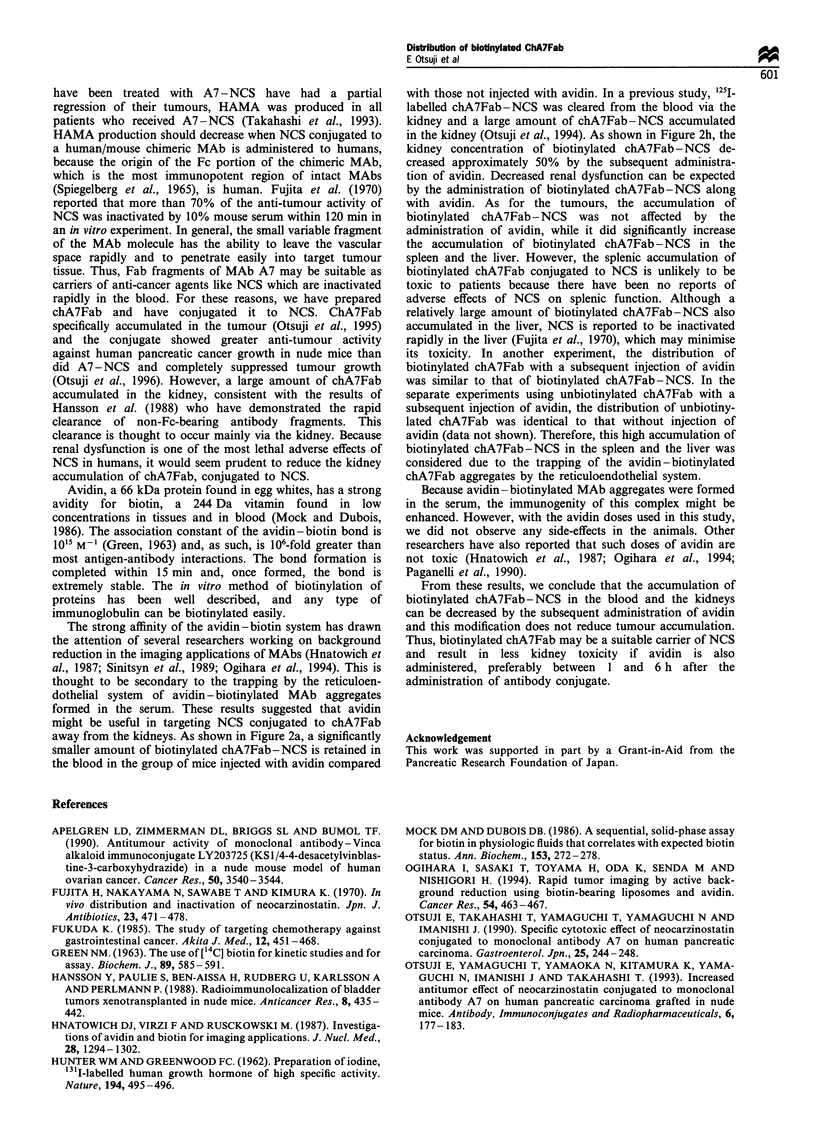

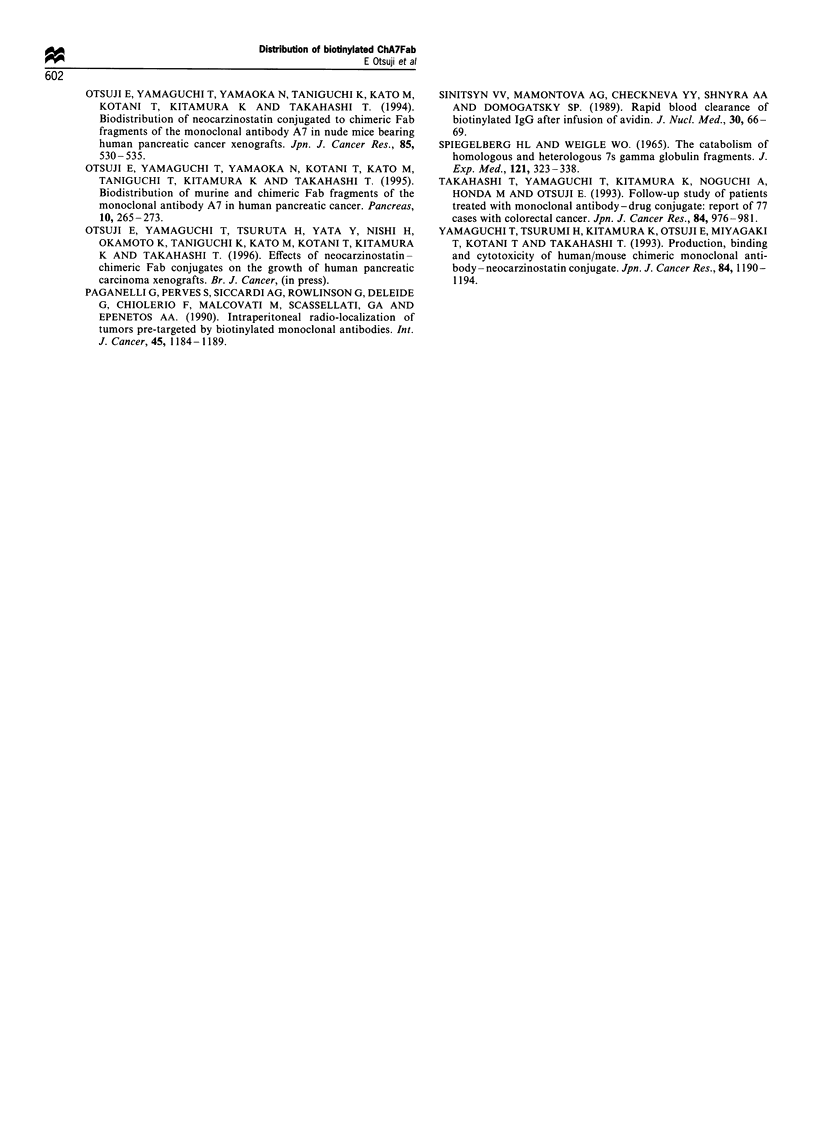


## References

[OCR_01017] Apelgren L. D., Zimmerman D. L., Briggs S. L., Bumol T. F. (1990). Antitumor activity of the monoclonal antibody-Vinca alkaloid immunoconjugate LY203725 (KS1/4-4-desacetylvinblastine-3-carboxhydrazide) in a nude mouse model of human ovarian cancer.. Cancer Res.

[OCR_01026] Fujita H., Nakayama N., Sawabe T., Kimura K. (1970). [In vivo distribution and inactivation of neocarzinostatin].. Jpn J Antibiot.

[OCR_01035] GREEN N. M. (1963). AVIDIN. 1. THE USE OF (14-C)BIOTIN FOR KINETIC STUDIES AND FOR ASSAY.. Biochem J.

[OCR_01048] HUNTER W. M., GREENWOOD F. C. (1962). Preparation of iodine-131 labelled human growth hormone of high specific activity.. Nature.

[OCR_01037] Hansson Y., Paulie S., Ben-Aïssa H., Rudberg U., Karlsson A., Perlmann P. (1988). Radioimmunolocalisation of bladder tumors xenotransplanted in nude mice.. Anticancer Res.

[OCR_01045] Hnatowich D. J., Virzi F., Rusckowski M. (1987). Investigations of avidin and biotin for imaging applications.. J Nucl Med.

[OCR_01055] Mock D. M., DuBois D. B. (1986). A sequential, solid-phase assay for biotin in physiologic fluids that correlates with expected biotin status.. Anal Biochem.

[OCR_01061] Ogihara-Umeda I., Sasaki T., Toyama H., Oda K., Senda M., Nishigori H. (1994). Rapid tumor imaging by active background reduction using biotin-bearing liposomes and avidin.. Cancer Res.

[OCR_01064] Otsuji E., Takahashi T., Yamaguchi T., Yamaguchi N., Imanishi J. (1990). Specific cytotoxic effect of neocarzinostatin conjugated to monoclonal antibody A7 on human pancreatic carcinoma.. Gastroenterol Jpn.

[OCR_01091] Otsuji E., Yamaguchi T., Yamaoka N., Kotani T., Kato M., Taniguchi K., Kiyamura K., Takahashi T. (1995). Biodistribution of murine and chimeric Fab fragments of the monoclonal antibody A7 in human pancreatic cancer.. Pancreas.

[OCR_01086] Otsuji E., Yamaguchi T., Yamaoka N., Taniguchi K., Kato M., Kotani T., Kitamura K., Takahashi T. (1994). Biodistribution of neocarzinostatin conjugated to chimeric Fab fragments of the monoclonal antibody A7 in nude mice bearing human pancreatic cancer xenografts.. Jpn J Cancer Res.

[OCR_01107] Paganelli G., Pervez S., Siccardi A. G., Rowlinson G., Deleide G., Chiolerio F., Malcovati M., Scassellati G. A., Epenetos A. A. (1990). Intraperitoneal radio-localization of tumors pre-targeted by biotinylated monoclonal antibodies.. Int J Cancer.

[OCR_01120] SPIEGELBERG H. L., WEIGLE W. O. (1965). THE CATABOLISM OF HOMOLOGOUS AND HETEROLOGOUS 7S GAMMA GLOBULIN FRAGMENTS.. J Exp Med.

[OCR_01115] Sinitsyn V. V., Mamontova A. G., Checkneva Y. Y., Shnyra A. A., Domogatsky S. P. (1989). Rapid blood clearance of biotinylated IgG after infusion of avidin.. J Nucl Med.

[OCR_01123] Takahashi T., Yamaguchi T., Kitamura K., Noguchi A., Honda M., Otsuji E. (1993). Follow-up study of patients treated with monoclonal antibody-drug conjugate: report of 77 cases with colorectal cancer.. Jpn J Cancer Res.

[OCR_01129] Yamaguchi T., Tsurumi H., Kitamura K., Otsuji E., Miyagaki T., Kotani T., Takahashi T. (1993). Production, binding and cytotoxicity of human/mouse chimeric monoclonal antibody-neocarzinostatin conjugate.. Jpn J Cancer Res.

